# Spectral Resting-State EEG (rsEEG) in Chronic Aphasia Is Reliable, Sensitive, and Correlates With Functional Behavior

**DOI:** 10.3389/fnhum.2021.624660

**Published:** 2021-03-17

**Authors:** Sarah G. H. Dalton, James F. Cavanagh, Jessica D. Richardson

**Affiliations:** ^1^Department of Speech Pathology and Audiology, Marquette University, Milwaukee, WI, United States; ^2^Department of Psychology, University of New Mexico, Albuquerque, NM, United States; ^3^Department of Speech and Hearing Sciences, University of New Mexico, Albuquerque, NM, United States

**Keywords:** aphasia, EEG, resting-state, spectral, reliability

## Abstract

We investigated spectral resting-state EEG in persons with chronic stroke-induced aphasia to determine its reliability, sensitivity, and relationship to functional behaviors. Resting-state EEG has not yet been characterized in this population and was selected given the demonstrated potential of resting-state investigations using other neuroimaging techniques to guide clinical decision-making. Controls and persons with chronic stroke-induced aphasia completed two EEG recording sessions, separated by approximately 1 month, as well as behavioral assessments of language, sensorimotor, and cognitive domains. Power in the classic frequency bands (delta, theta, alpha, and beta) was examined via spectral analysis of resting-state EEG data. Results suggest that power in the theta, alpha, and beta bands is reliable for use as a repeated measure. Significantly greater theta and lower beta power was observed in persons with aphasia (PWAs) than controls. Finally, in PWAs theta power negatively correlated with performance on a discourse informativeness measure, while alpha and beta power positively correlated with performance on the same measure. This indicates that spectral rsEEG slowing observed in PWAs in the chronic stage is pathological and suggests a possible avenue for directly altering brain activation to improve behavioral function. Taken together, these results suggest that spectral resting-state EEG holds promise for sensitive measurement of functioning and change in persons with chronic aphasia. Future studies investigating the utility of these measures as biomarkers of frank or latent aphasic deficits and treatment response in chronic stroke-induced aphasia are warranted.

## Introduction

For over two million adults living with aphasia in the United States, effective neurorehabilitation will be critical to successful return to everyday life pursuits. Resting-state EEG (rsEEG) holds potential as a measure of neurorehabilitation success because it is inexpensive, has few contra-indications for use, and is already widely used in hospitals. Further, data acquisition is straightforward for trained users, even in individuals with severe impairments, and commercial systems with data processing applications are already positioned in healthcare settings, making it highly feasible.

Resting-state assessments offer a simple complement to task-based paradigms in the identification of functional network connectivity. While task-based paradigms provide important insights into behaviors, they can be difficult to interpret when completed by individuals with an impairment in the behavior of interest (Price et al., 2006). For example, investigations of language in persons with aphasia (PWAs) often require participants to name pictures or match pictures to a word while undergoing fMRI. Because these individuals have difficulty processing and producing language, incorrect responses are common and must be accounted for in paradigm design and subsequent analyses. Typically, this means excluding incorrect trials from analysis, at the cost of power to detect effects. To offset this loss of statistical power, more trials can be included, but at the risk of increasing fatigue, frustration, and emotional distress. Further, the cognitive load of a given task will vary greatly across participants, depending on their level of deficit and residual abilities (Logan, 1985; Borghini et al., 2017). These differences in cognitive load mean that recruitment of brain regions for task completion will also vary greatly, further complicating inferences about task-based activation. Similarly, event-related potential (ERP) analyses are used to investigate patterns in the EEG signal during task completion, with the same interpretive difficulties observed as in task-based fMRI. Recently, resting-state fMRI (rsfMRI) has allowed investigation of brain function in the absence of task-based behavior (Veldsman et al., 2014), which has led to an improved understanding of how networks communicate at rest and when on-task (Klingbeil et al., 2019). It is reasonable to expect that spectral analysis of rsEEG may offer similar benefits as rsfMRI, enabling assessment of PWAs even when language deficits limit or prevent completion of tasks that rely on intact language systems.

Spectral rsEEG in the acute and sub-acute stages following stroke reveals increased low frequency (delta and theta) activity and reduced high frequency (alpha and beta) activity compared to controls (for a review, see Finnigan and van Putten, 2013). These differences between controls and PWAs seem to be related to functional outcomes as measured by general stroke scales (Finnigan et al., 2007; de Vos et al., 2008; Sheorajpanday et al., 2009; Dubovik et al., 2013; Nicolo et al., 2015), where increased low frequency activity and reduced high frequency activity is often associated with poorer outcomes. Only a few studies have examined spectral EEG in chronic stroke (Rozelle and Budzynski, 1995; Spironelli and Angrilli, 2009; Spironelli et al., 2013; Herron et al., 2014; Song et al., 2015). The utility and generalizability of these studies are limited due to their reliance on behavioral tasks, restricted spectral frequencies investigated, and restricted behavioral profiles (e.g., persons with Broca’s aphasia only). While completing language tasks, including lexical judgment, rhyming judgment, semantic matching, and orthographic matching, 17 Italian-speaking PWAs (3 non-fluent, 13 fluent, 1 unclassified) demonstrated increased delta power in the left hemisphere compared to healthy controls (Spironelli and Angrilli, 2009). In a related study with an overlapping sample, Spironelli et al. (2013) reported that 11 Italian-speaking PWAs also had reduced high beta power (calculated by averaging activity in the 21–28 Hz range, the upper half of the classic beta frequency band) in the lesioned left hemisphere compared to controls.

While not always specific to PWAs, several investigations have examined changes in spectral EEG over time. Spectral task-based EEG in 11 German-speaking PWAs (five non-fluent, six fluent) demonstrated that decreased left hemisphere delta power corresponded to significant language recovery in the first year post stroke, but no changes in language recovery or delta power were seen in the second year post stroke (Hensel et al., 2004). In a similar study of cognitive function (measured by the MoCA) following mild right hemisphere stroke in 10 Serbian-speaking participants, Petrovic et al. (2017) reported that spectral rsEEG features measured approximately 10 days post-stroke did not fully resolve 1–1.5 years post-stroke, even when cognitive behavioral performance did. It was posited that these permanent changes in rsEEG were a result of neural adaptations to support cognition in the face of a lesion.

Some researchers have also examined spectral EEG before and after rehabilitation in sub-acute and chronic PWAs (Rozelle and Budzynski, 1995; Stojanovic et al., 2013). A single case study of an English-speaking person with chronic non-fluent aphasia investigated the use of spectral EEG as a biofeedback method to lower theta and increase beta activity (Rozelle and Budzynski, 1995). Following training, the participant demonstrated significantly reduced theta activity in spectral rsEEG, alongside behavioral improvements in speech, language, motor, mood, and cognitive domains. More recently, a study investigated aphasia treatment response in a sample of 32 Serbian-speaking PWAs (26 non-fluent, 6 fluent) using spectral rsEEG to compare the hemispheric and regional symmetry of delta, theta, alpha and beta power (Stojanovic et al., 2013). Prior to treatment, hemispheric and regional asymmetry were increased, and variability was decreased, in PWAs compared to 86 age- and sex-matched healthy controls. Following treatment, between-group differences were significantly decreased, driven primarily by the better responders to aphasia therapy.

Taken together, these studies provide preliminary evidence that spectral rsEEG may be useful for prognosis and measurement of treatment response. However, the psychometric properties of spectral rsEEG have not yet been characterized in PWAs, limiting the validity and accuracy of predictive inferences that can be made. A necessary step in the translation of spectral rsEEG measures from research to clinical practice is ensuring they possess adequate psychometric properties for use as repeated measures (i.e., treatment monitoring). Thus, it is paramount that the psychometric properties of spectral rsEEG are defined in PWAs, particularly in the chronic stage, as this has been understudied in comparison to the acute and sub-acute stages. Investigations of spectral EEG variability in adults have generally demonstrated good stability for healthy populations (Oken and Chiappa, 1988; Salinsky et al., 1991; Corsi-Cabrera et al., 1997; Kondacs and Szabó, 1999; Gudmundsson et al., 2007; Suarez-Revelo et al., 2015), though electrode montage (i.e., spatial arrangement of electrodes) can have a significant impact on reliability (Gudmundsson et al., 2007), warranting further investigation. Acceptable specificity and test-retest reliability has yet to be investigated or established for specific patient populations. Despite this very limited psychometric evidence to support the use of spectral EEG as a repeated measure, it is already being used in this manner in research (e.g., Rozelle and Budzynski, 1995; Hensel et al., 2004; Stojanovic et al., 2013; Wu et al., 2015; Petrovic et al., 2017). Defining specificity and reliability of spectral rsEEG measures utilizing varied electrode montages post-stroke is critical to ensuring appropriate application of these measures and preventing research waste (for a discussion of research waste as it pertains to biomarkers, see Ioannidis and Bossuyt, 2017).

The current study seeks to improve our understanding of brain activation changes and their potential as a biomarker (i.e., indicator of presence of aphasia, indicator of treatment response) in persons with chronic stroke-induced aphasia by repeated examination of the four most frequently used spectral EEG frequency bands during two rest conditions (eyes-open and eyes-closed). We focus our investigation on the chronic stage for two reasons. First, spectral rsEEG is understudied in the chronic phase compared to acute and sub-acute stages; and second, persons with chronic aphasia are likely to have more stable brain functions, making this the ideal population for investigating spectral rsEEG’s appropriateness for repeated measurement. This study will provide confirmation of spectral rsEEG changes persisting into the chronic stage post-stroke and will have wide application to PWAs of varying severities, where accurate completion of language tasks may not be possible. We report results for varied montages given the reported differences in the reliability of montages, the use of varied montages in the literature, and the contrast between clinically focused research which uses fewer electrodes (typically no more than 19) versus laboratory-focused research which uses large numbers of electrodes (64–256). The specific aims of this study are to:

1. Determine the reliability of spectral rsEEG measures in healthy controls and PWAs for four montages in order to establish suitability as a repeated measure.
2. Examine differences in spectral rsEEG between well-described samples of healthy controls and PWAs.
3. Examine relationships between spectral rsEEG measures and performance on behavioral measures.

## Materials and Methods

### Participants

#### Persons With Aphasia

Twenty-one PWAs were recruited into the study. Following inspection of EEG data, two participants were removed from analysis due to poor data quality, leaving 19 (seven females) PWAs (Table 1). Participants who had experienced multiple strokes (2–4) were included to ensure that the results are maximally applicable to the general aphasia rehabilitation population served by speech-language pathologists. Fifteen participants experienced left hemisphere stroke(s) while four experienced stroke(s) of mixed hemisphericity. Western Aphasia Battery - Revised (WAB-R; Kertesz, 2006) Aphasia Quotient (AQ) scores were used to classify participants into the following aphasia subtypes: 6 anomic, 3 conduction, 1 Wernicke’s, 1 Broca’s, 1 transcortical motor, and 7 who experienced left hemisphere strokes but tested above the WAB cut-off for clinical aphasia (not aphasic by WAB; NABW). PWAs NABW demonstrated communication deficits based on discourse and naming performance (described in detail below) and were therefore included in the study, consistent with previous research (Spironelli and Angrilli, 2009; Spironelli et al., 2013; Fromm et al., 2017; Dalton and Richardson, 2019). All PWAs were greater than 1-year post-stroke to ensure that spontaneous recovery was not a factor in the reliability analysis. As in previous studies (e.g., Finnigan et al., 2007, 2016; Leon-Carrion et al., 2009; Herron et al., 2014; Nicolo et al., 2015; Song et al., 2015; Wu et al., 2015; Gorišek et al., 2016), potential participants with a diagnosis of significant psychiatric mood disorders were excluded (including major depressive disorder or generalized anxiety disorder), but persons with self-reported symptoms of mild depression and anxiety with no clinical diagnosis were allowed to participate. All PWAs were right-handed prior to their stroke. English was the primary language used by all participants at the time of testing, and had been for many years, according to responses on the Language Experience and Proficiency Questionnaire (LEAP-Q; Marian et al., 2007). Three participants with stroke reported speaking more than one language, and all but one participant (who reported Spanish as the first language) reported English as their first language. The average age of PWAs was 58.2 years (SD = 14.5). Average education was 15 years (SD = 3.1). See Supplementary Table 1 for participant level demographics of PWAs, including available lesion location information.

**TABLE 1:** Demographics for neurologically healthy controls and persons with aphasia included in the analysis.

	**Healthy controls**
Sex	15 females; 9 males
Age (years)	59.3 (15.1) *Range: 22–79*
Education (years)	16.8 (2.7) *Range: 12–22*
Bi/Multilingual	6

	**Persons with aphasia**

Sex	7 females; 12 males
Age (years)	58.2 (14.5) *Range: 33–87*
Education (years)	15 (3.1) *Range: 7–20*
Bi/Multilingual	2
Number of strokes	1.4 (0.8) *Range: 1–4*
Time post onset in months (since most recent stroke)	54.2 (40.8) *Range: 15–183*
Lesioned hemisphere	15 left; 4 mixed
Aphasia diagnosis	7 NABW 6 Anomic 3 Conduction 1 Wernicke’s 1 Transcortical motor 1 Broca’s
Cognitive deficit^†^	12
Sensorimotor deficit^†⁣†^	16

*Values for non-count variables are reported as mean (standard deviation). ^†^One participant did not complete RBANS testing, for this participant determination of cognitive deficit is based on performance on the Weschler Adult Intelligence Scales Picture Completion subtest. ^†⁣†^The same participant did not complete the sensorimotor assessment, however, the participant had hemiparesis observable in everyday activities.*

#### Healthy Controls

Twenty-six control participants were recruited into the study. Following inspection of EEG data, two participants were removed from analysis, leaving 24 (15 females) healthy, native English-speaking controls (Table 1). Participants were screened to ensure no history of neurological disease or injury that might affect brain function. Potential participants with a diagnosis of significant psychiatric mood disorders were excluded (including major depressive disorder or generalized anxiety disorder), but persons with self-reported symptoms of mild depression and anxiety with no clinical diagnosis were allowed to participate. All healthy control participants were right-handed, and the majority were monolingual; six reported speaking at least one other language in addition to English, but English was the first language for all control participants according to LEAP-Q responses (Marian et al., 2007). Healthy controls were matched to PWAs primarily on age, with years of education as a secondary matching variable. For one PWA, we were unable to match both age and education, due to the low level of education (seventh grade level) attained. The average age of control participants was 59.3 years (*SD* = 15.1 years). Control participants averaged 16.8 years of education (*SD* = 2.7).

### Assessments

In order to fully describe the characteristics of the sample, all participants (PWAs and healthy controls) completed sensorimotor, cognitive, and speech-language testing (Table 2). Sensorimotor testing was included since many individuals with aphasia experience these deficits given the close proximity of primary and supplementary motor cortices to perisylvian language areas. Sensorimotor function was measured via an in-house battery that included sensation, proprioception, motricity, and fine motor coordination tasks. Cognitive function was assessed with the Repeatable Battery for the Assessment of Neuropsychological Status (RBANS; Randolph, 1998) and the Wechsler Adult Intelligence Scales - Picture Completion subtest (WAIS-PC; Wechsler et al., 2008). Motor speech function was assessed using subtests of the Apraxia Battery for Adults - 2 (ABA-2; Dabul, 2000). Processing and production of prosodic information in spoken language was assessed using the Aprosodia Battery (Ross and Monnot, 2011). Narrative abilities were assessed with the Discourse Production Test (DPT; Fromm et al., 2020), which requires participants to tell a story depicted in pictures, retell the story of Cinderella, and describe how to make a peanut-butter and jelly sandwich. Additional language measures were administered to PWAs only and included the WAB-R (Kertesz, 2006) to assess overall aphasia severity, the Boston Naming Test (BNT; Kaplan et al., 2001) to assess anomia severity, and a shortened version of the Discourse Comprehension Test (DCT; Brookshire and Nicholas, 1997).

**TABLE 2:** Test scores for healthy controls and persons with aphasia included in the analysis as well as Cohen’s *D* effect sizes to estimate the magnitude of the difference between groups.

			**Control**	**PWA**	**Cohen’s *D***
**Sensorimotor**	Sensory	Palm sensation (2)	2 (0)	1.6 (0.6)	0.897
		Proprioception (11)	11 (0.2)	9.7 (1.9)	0.988
	Range of motion (ROM); Strength	Brunstromm left hand function (6)	6 (0)	5.9 (0.3)	0.393
		Brunstromm right hand function (6)	6 (0)	4.9 (1.8)	0.990
		Left motricity index (199)	197.1 (3)	172.1 (48.5)	0.765
		Right motricity index (199)	197.4 (2)	146.7 (56.7)	1.331
	Fine motor; Coordination	Left index finger tap	50.3 (8)	42.4 (12.6)	0.769
		Right index finger tap	53.9 (8.8)	33.4 (22.1)	1.279
		L index/middle finger tap	50 (18.8)	45.7 (25.2)	0.196
		R index/middle finger tap	55.7 (19.7)	33.7 (27.5)	0.939
		L/R index finger tap	66.45 (17.4)	40.5 (28.4)	1.123
		L foot tap	32.6 (7)	31.4 (8)	0.165
		R foot tap	36 (7.6)	25.8 (11.8)	1.060
		L/R foot tap	47.6 (10.3)	35.9 (17.5)	0.840

**Cognitive**		RBANS	97.2 (13.9)	64.7 (16.9)	2.130
		WAIS - PC	13.4 (2.8)	9.3 (3.6)	1.284

**Emotion**	Aprosodia battery-Reception-Expression	Word ID (12)	10.9 (1.3)	8.2 (2.8)	1.263
		Monosyllabic ID (12)	10.2 (1.1)	8 (2.2)	1.303
		Asyllabic ID (12)	9.1 (2.2)	6.5 (2.7)	1.079
		Facial expression (14)	12.7 (1.2)	10.5 (2.3)	1.174
		Verbal scenario (14)	12.6 (1.4)	10.3 (2.8)	1.049
		Attitude (20)	16.7 (2)	14.6 (2.6)	0.907
		Emotion semantics (20)	19.8 (0.4)	17.9 (2.4)	1.113

**Language**	Main concept analysis	MC composite (216)	128.7 (21.8)	90.2 (44.5)	1.127
		Accurate/complete (72)	35.4 (7.7)	22.4 (14.2)	1.160
	Motor speech	Increasing length	0.1 (0.2)	3.2 (3.1)	−1.482
		Limb/oral apraxia (50)	50 (0)	47.3 (3)	1.345
		Multiple repetition (30)	29.9 (0.5)	25.3 (7.8)	0.878
		WAB-R-AQ (100)		83.9 (16.9)	
		BNT (60)		44.5 (15.2)	
	Discourse comprehension	Main idea – Total (12)		10.9 (1.7)	
		Detail – Total (12)		9.2 (2.1)	

*The value next to each task name represents the maximum score on the measure. Performance on each task is reported as mean (standard deviation). All effect sizes, except those for left hand function, left index/middle finger tap, and left foot tap, correspond to large effects. RBANS, Repeatable Battery for the Assessment of Neuropsychological Status; WAIS-PC, Weschler Adult Intelligence Scales - Picture Completion; WAB-R-AQ, Western Aphasia Battery - Revised - Aphasia Quotient; BNT, Boston Naming Test.*

The DPT was scored using a main concept analysis (MCA; Nicholas and Brookshire, 1995), which evaluates how well persons communicate the gist, or essential elements of a story. MCA was scored using standardized checklists with accompanying normative data (Nicholas and Brookshire, 1995; Richardson and Dalton, 2016, 2020; Dalton and Richardson, 2019) which involves comparison of participant-produced utterances to standardized checklists to evaluate accuracy and completeness of utterances. For example, during a retelling of the Cinderella story, one of the main concepts is “Cinderella danced with the prince” (with three essential elements ‘Cinderella,’ ‘danced,’ and ‘with the prince’). If an individual attempted to produce this main concept and said, “Cinderella danced”, it would be considered accurate but incomplete, because it is missing the essential element ‘with the prince.’ If an individual said “Cinderella walked with the prince” it would be considered inaccurate (because they dance, not walk) but complete, because all three essential elements are represented. A score is assigned for each main concept ranging from 0 for absent to 3 for accurate and complete and then scores are summed to yield the MC composite score. In this investigation we focused on the MC composite score and the number of MCs coded as accurate and complete since they are the most straightforward to interpret. Using this system, we can sensitively assess the quality of discourse production in individuals across the impairment spectrum, from healthy controls (e.g., no history of stroke and aphasia) to profound aphasia (Dalton and Richardson, 2019; Dalton et al., 2020).

### EEG Recording

EEG data was recorded from 64 active electrodes placed in an elastic cap according to the 10–10 International system of classification (Chatrian et al., 1985) which extends the 10–20 placement system for more dense electrode arrays. The ground electrode was located at Fpz (the default in the electrode caps used in this study) with the reference electrode at CPz. Blinks and eye movement were recorded via vertical electro-oculography using paired electrodes placed above and below the left eye. Data were recorded on a BrainVision actiCHamp system with a 500 Hz sampling rate (corresponding to a data point recorded every 2 ms) and online bandpass filtering from 0.01–100 Hz. Participants were seated in front of a computer in a dimly lit room. rsEEG was recorded for 2 min with eyes-open and 2 min with eyes-closed. During the eyes-open recording, participants were asked to fixate on a white cross presented on a black background to limit eye movement artifacts. Both eyes-open and eyes-closed conditions were used as there is inconsistency in the literature, with most reporting only the eyes-closed condition (e.g., Dubovik et al., 2013; Finnigan et al., 2016), others reporting only the eyes-open condition (e.g., Herron et al., 2014; Wu et al., 2015), and some reporting both conditions (e.g., Cuspineda et al., 2003; Stojanovic et al., 2013). In addition, research indicates that these conditions are not equivalent, even as individuals age, suggesting a need to determine psychometric properties for each condition separately (Barry et al., 2007; Barry and De Blasio, 2017).

### Data Processing

Standard offline pre-processing using BrainVision Analyzer 2.1 was conducted to ensure adequate data quality. First, noisy channels were identified through visual inspection and interpolated. Data were high (0.1 Hz) and low (50 Hz) pass filtered using infinite impulse response zero-phase shift Butterworth filters to minimize distortion and preserve phase information (Hamming, 1998; Oppenheim et al., 1999). After filtering, bad segments (i.e., muscle activity) were manually rejected and independent component analysis was conducted to remove eye movement artifacts (Makeig et al., 1996). Channels were re-referenced to an average reference and channel CPz was interpolated from the average referenced data.

After the pre-processing steps above, the following steps were conducted to calculate absolute spectral power. First, data were epoched into 2048 ms bins with 1024 data points per epoch. Epochs with data values greater than ±100 microvolts and/or changes in value greater than ±25 microvolts were rejected. Second, data were subjected to a fast Fourier transform (FFT) with Hanning window and tapering at the beginning and end of the window totaling 10% of the epoch length. The resolution of a FFT can be calculated as the sampling frequency divided by the number of data points in the epoch. With a sampling frequency of 500 Hz and 1024 data points in each epoch, this resulted in 0.49 Hz resolution, consistent with previous research (e.g., Hensel et al., 2004; Herron et al., 2014; Schleiger et al., 2014). Participants with fewer than 30 s of artifact-free data per condition were excluded from the analysis, resulting in the exclusion of data for two PWAs and two healthy controls. Third, the absolute sum of spectral power in the four classic frequency bands was calculated, accounting for the 0.49 Hz resolution of the FFT (delta: 0.98–2.93; theta: 3.91–6.84 Hz; alpha: 7.81–12.21 Hz; beta: 13.18–30.27 Hz). These frequency bands were selected based on the most commonly reported values in the articles we reviewed (e.g., Leon-Carrion et al., 2009; Herron et al., 2014). All measures were calculated for each electrode separately, then averaged across electrodes, a common approach in the literature (e.g., Cuspineda et al., 2003; Hensel et al., 2004; Finnigan et al., 2007; Song et al., 2015; Carrick et al., 2016).

**FIGURE 1:**
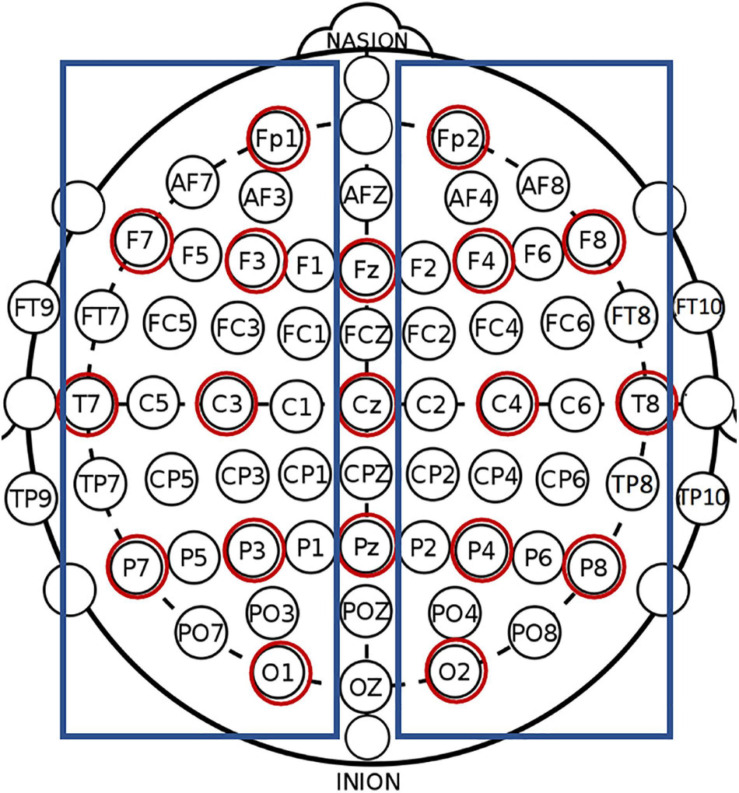
All labeled electrodes were included in the whole brain montage. Red circles show electrodes included the clinical (10–20 montage), and blue boxes show electrodes included in the left and right hemisphere montages.

Calculations were performed for the following anatomical electrode montages (see Figure 1): whole brain (all 64 electrodes), clinical (19 electrodes corresponding to 10–20 International system electrode locations; e.g., Finnigan et al., 2016), left hemisphere (excluding midline electrodes), and right hemisphere (excluding midline electrodes). For the left and right hemisphere montages, the average reference was calculated separately using only the electrodes in that hemisphere to ensure that only activation in the hemisphere of interest was included. Previous research has reported on hemispheric comparisons in both healthy controls and individuals post-stroke, indicating a need for information regarding the stability and reliability of such measures (e.g., Hensel et al., 2004; Spironelli and Angrilli, 2009; Spironelli et al., 2013; Herron et al., 2014; Petrovic et al., 2017). Also, neuroimaging studies have long utilized hemispheric comparisons and/or examined hemispheric differences and related behaviors (e.g., French and Beaumont, 1984; Bolduc et al., 2003; Szaflarski et al., 2006; Learmonth et al., 2017; Othman et al., 2020). Hemispheric comparisons are frequent in the aphasia literature where lesions affect a behavior (e.g., language) that is typically strongly lateralized to the left hemisphere (e.g., Gow and Ahlfors, 2017; Piai et al., 2017; Sandberg, 2017; Wilson et al., 2019). By providing hemispheric results in this investigation, we pave the road for future investigations that seek to compare individuals with left and right hemisphere strokes, or to compare individuals with either left or right hemisphere strokes to healthy controls or other clinical populations.

### Data Analysis

All statistical analyses were conducted using SPSS v27. The reliability of spectral rsEEG absolute power was calculated between sessions one and two via single-measure intra-class correlations using a two-way mixed effect model with absolute agreement (ICC; Shrout and Fleiss, 1979; McGraw and Wong, 1996; Suarez-Revelo et al., 2015; Koo and Li, 2016). ICCs are widely used to evaluate the psychometric properties of newly developed assessment instruments. The ICCs conducted here assessed the exactness of the match between, for example, whole brain relative delta power at session one and whole brain relative delta power at session two during eyes-closed rest. The closer these values are to each other, the stronger the correlation, and the more stable the measure over time. Only the point estimates are reported in the text; however, 95% confidence intervals are reported in tables to allow readers a more nuanced interpretation of reliability. ICCs were identified as poor (<0.5), moderate (≥0.5 and <0.75), good (≥0.75 and <0.9), or excellent (≥0.9) (Koo and Li, 2016).

Before examining between-group differences, descriptive statistics (mean, median, standard deviation, range, skew, and kurtosis) were calculated for both groups; normality was assessed using skew and kurtosis. Student’s *t*-tests to compare differences between groups were planned. While many variables reported here violate the assumption of normality, previous research has shown parametric statistics such as the *t*-test to be robust to violations of normality (using Bradley’s [1978] definition of robustness where deviation from *p* = 0.05 is ∼±0.005). Simulation studies have demonstrated the robustness of the *t*-test in the face of non-normal distributions when the absolute value of skew is less than 2, and the absolute value of kurtosis is less than 9 (Boneau, 1960; Posten, 1978; Bradley, 1982; Schmider et al., 2010). Data with skew or kurtosis outside the range for which *t*-tests are robust were assessed using the Mann–Whitney *U* test consistent with previous research (Hensel et al., 2004; Nolfe et al., 2006; Sheorajpanday et al., 2009; Herron et al., 2014). Homogeneity of variance was assessed using the Levene’s test. For variables that violated the homogeneity of variance assumption, Welch’s *t*-tests were used (Ruxton, 2006). Between-group comparisons were conducted using the data from the first recording session only. Effect size calculations (Hedge’s *g* for *t*-tests and η^2^ for Mann–Whitney *U* tests) were conducted for all comparisons, and medium to large effects are reported. Hedge’s *g* and η^2^ have a different range of possible values, so for ease of interpretation, both the numeric value and commonly accepted estimates of effect size (small, medium, or large) are reported (Cohen, 1988). Holm–Bonferroni corrections for multiple comparisons (Holm, 1979) were used to control type I error. We selected the Holm–Bonferroni approach over the more widely used Bonferroni technique as the Bonferroni tends to inflate Type I errors, while the Holm–Bonferroni approach better balances Type I and Type II error (Aickin and Gensler, 1996), and will be more sensitive to group differences.

Finally, an analysis of correlations between spectral rsEEG measures and performance on behavioral tasks in PWAs was conducted. Correlations were calculated to identify the relationship between spectral rsEEG measures and performance on cognitive (RBANS index score), and language (WAB-R AQ, number of correct items named on BNT, and production of Main Concepts [MCs] during storytelling) assessments. For all behavioral measures, a lower score represents poorer performance.

## Results

Independent samples *t*-tests were conducted to compare age and education between the two groups to ensure adequate matching. No significant difference was observed between the two groups for age (*t* = 0.239; *p* = 0.812). A significant difference was observed between groups on education (*t* = 2.081; *p* = 0.044), driven by the PWA with a seventh grade education. When this individual was removed from the PWA group, the *t*-test for education was no longer significant (*t* = 1.715; *p* = 0.094).

### Aim 1 - Reliability

Reliability was examined for each of the four montages (whole brain, clinical, left hemisphere, right hemisphere) in each spectral frequency band (delta, theta, alpha, beta). This resulted in the calculation of ICCs for 16 montage + spectral band combinations.

#### Eyes-Open Rest

**FIGURE 2:**
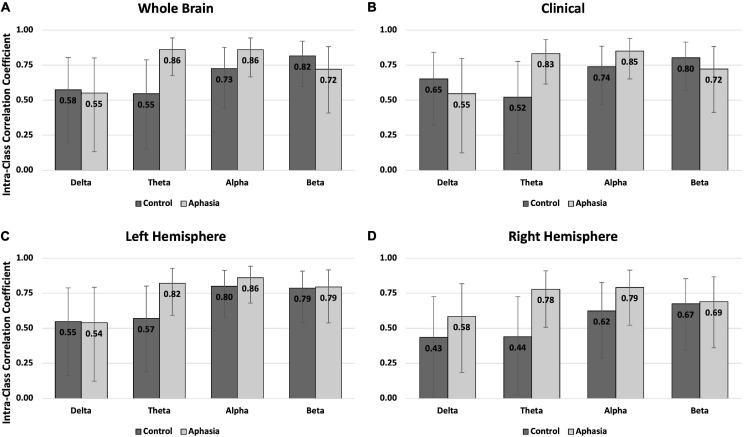
Intra-class correlations by group and frequency band for the eyes open rest condition in **(A)** the whole brain montage; **(B)** the clinical montage; **(C)** the left hemisphere montage; and **(D)** the right hemisphere montage. Error bars represent the 95% confidence interval of the point estimates reported in Table 3.

Better intra-individual reliability was observed for PWAs than controls during eyes-open rest (Table 3 and Figure 2). PWAs demonstrated moderate-good reliability in all montage + spectral band combinations (good reliability in 9/16 and moderate reliability in 7/16), while healthy controls demonstrated moderate-good reliability in 14/16 montage + spectral band combinations (good reliability in 4/16 and moderate reliability in 10/16). Healthy controls demonstrated poor reliability for delta and theta power in the right hemisphere. For PWAs, the highest reliability was observed with theta and alpha power, then beta power, with the lowest reliability in delta power. In healthy controls the highest reliability was observed with beta power, followed by alpha, then delta and finally theta power.

**TABLE 3:** Intra-class correlation coefficients and 95% confidence intervals for neurologically healthy controls and persons with aphasia.

	**Control**									
			
	**Eyes open rest**					**Eyes closed rest**				
	**Delta**	**Theta**	**Alpha**	**Beta**	**Average**	**Delta**	**Theta**	**Alpha**	**Beta**	**Average**
Whole brain	0.575	0.546	0.725	0.815	*0.665*	0.566	0.878	0.727	0.750	*0.73*
	0.197–0.804	0.152—-0.788	0.444–0.877	0.597–0.921		0.179–0.799	0.727–0.948	0.437–0.880	0.484–0.890	
Clinical	0.652	0.522	0.738	0.802	*0.679*	0.608	0.852	0.704	0.742	*0.727*
	0.322–0.842	0.119–0.776	0.467–0.884	0.572–0.915		0.241–0.821	0.669–0.937	0.397–0.869	0.467–0.886	
Left hemisphere	0.548	0.570	0.800	0.786	*0.676*	0.469	0.746	0.652	0.655	*0.631*
	0.164–0.788	0.190–0.801	0.576–0.913	0.541–0.908		0.045–0.746	0.471–0.889	0.309–0.843	0.314–0.845	
Right hemisphere	0.434	0.439	0.624	0.674	*0.543*	0.602	0.782	0.790	0.778	*0.738*
	0.003–0.726	0.024–0.726	0.286–0.826	0.346–0.854		0.241–0.817	0.537–0.906	0.550–0.90	0.532–0.904	
Average	*0.552*	*0.519*	*0.721*	*0.769*		*0.561*	*0.815*	*0.718*	*0.731*	
	**Persons with aphasia**									
			
	**Eyes open rest**					**Eyes closed rest**				
	**Delta**	**Theta**	**Alpha**	**Beta**	**Average**	**Delta**	**Theta**	**Alpha**	**Beta**	**Average**

Whole brain	0.551	0.861	0.860	0.721	*0.748*	0.626	0.807	0.817	0.667	*0.729*
	0.132–0.801	0.674–0.944	0.666–0.944	0.409–0.882		0.255–0.837	0.564–0.921	0.583–0.926	0.331–0.856	
Clinical	0.546	0.832	0.851	0.722	*0.738*	0.593	0.793	0.792	0.665	*0.711*
	0.125–0.798	0.615–0.932	0.652–0.940	0.413–0.883		0.195–0.822	0.535–0.915	0.533–0.915	0.327–0.855	
Left hemisphere	0.539	0.821	0.860	0.794	*0.754*	0.491	0.819	0.796	0.714	*0.705*
	0.123–0.793	0.592–0.927	0.679–0.943	0.539–0.916		0.061–0.767	0.596–0.926	0.540–0.916	0.404–0.878	
Right hemisphere	0.584	0.778	0.791	0.689	*0.711*	0.768	0.734	0.695	0.674	*0.604*
	0.185–0.817	0.507–0.909	0.522–0.915	0.360–0.867		0.490–0.904	0.437–0.888	0.369–0.870	0.339–0.859	
Average	*0.555*	*0.823*	*0.841*	*0.719*		*0.62*	*0.788*	*0.775*	*0.68*	

*The average reliability for electrode montages and frequency band is reported in the columns and rows labeled “average”. Cells are color-coded according to the guidelines reported by Koo and Li (2016) as poor (red), moderate (yellow), and good (green).*

#### Eyes-Closed Rest

**FIGURE 3:**
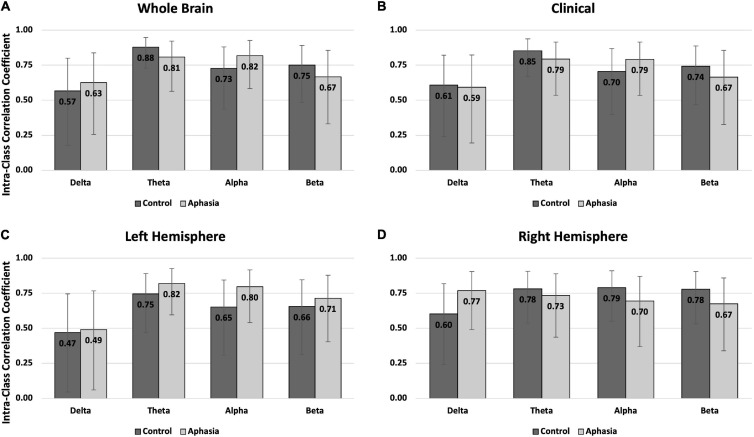
Intra-class correlations by group and frequency band for the eyes closed rest condition in **(A)** the whole brain montage; **(B)** the clinical montage; **(C)** the left hemisphere montage; and **(D)** the right hemisphere montage. Error bars represent the 95% confidence interval of the point estimates reported in Table 3.

Reliability in the eyes-closed condition was similar across groups, with better reliability for PWAs than controls (Table 3 and Figure 3). For PWAs, moderate-good reliability was demonstrated in 15/16 montage + spectral band combinations (good reliability in 7/16 and moderate reliability in 8/16). Controls also demonstrated moderate-good reliability in 15/16 montage + spectral band combinations (good reliability in 5/16 and moderate reliability in 10/16). Both groups demonstrated poor reliability of delta power in the left hemisphere. For PWAs, highest reliability was observed for theta and alpha power, then beta, and finally delta. For healthy controls, highest reliability was observed for theta power, followed by alpha and beta, and finally delta power.

### Aim 2 - Group Differences

**FIGURE 4:**
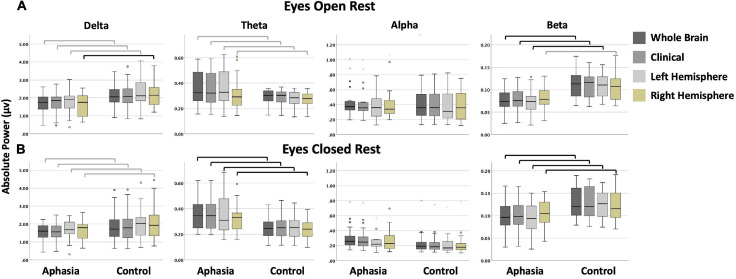
Boxplots display the absolute power (in microvolts) of each frequency band by group and montage for **(A)** the eyes open; and **(B)** the eyes closed condition. Black bars indicate statistically significant comparisons with large effects (corresponding to bolded cells in Table 5). Gray bars indicate comparisons that were not significant after corrections for multiple comparisons but that had medium to large effect sizes (corresponding to italicized cells in Table 5). (In a boxplot, data are split into quartiles and the figure attributes are as follows: the top of the box represents the third quartile; the bottom of the box represents the first quartile; the length of the box from the top to the bottom represents the interquartile range; the horizontal line within the box is the median of the dataset; the upper whisker is the line from the top of the box to the maximum value, or in the presence of an outlier, 1.5 times the interquartile range above the third quartile; the lower whisker is the line from the bottom of the box to the minimum value, or in the presence of an outlier, to 1.5 times the interquartile range below the first quartile; circles and stars represent outliers, defined as any point beyond 1.5 times the interquartile range either above the third quartile or below the first quartile).

A repeated measures ANOVA was conducted to investigate potential differences in spectral rsEEG over time. The results of this analysis revealed that neither the main effect [*F*(1,1278) = 0.130, *p* = 0.718] nor the interaction term [*F*(1,1278) = 1.267, *p* = 0.261] were statistically significant, indicating that spectral power in the first and second session did not significantly differ. For this reason, only data from the first session was used in the following analyses. Descriptive statistics for both groups are reported in Table 4, with boxplots providing a visual representation of the data in Figure 4. Examination of skew and kurtosis values indicated that parametric *t*-tests were acceptable for all montages and frequencies except alpha power during the eyes-open condition (please see Supplementary Table 2 for skew and kurtosis values for all measures). Therefore, Mann–Whitney *U* tests were conducted for alpha power comparisons.

**TABLE 4:** Mean (top number) and standard deviation (bottom number) of frequency bands in each montage during eyes open and eyes closed rest for controls and PWAs.

	**Control**							
	**Eyes open rest**				**Eyes closed rest**			
	**Delta**	**Theta**	**Alpha**	**Beta**	**Delta**	**Theta**	**Alpha**	**Beta**
Whole brain	2.165	0.286	0.231	0.127	1.885	0.253	0.404	0.111
	0.647	0.064	0.141	0.034	0.845	0.081	0.192	0.029
Clinical	2.192	0.284	0.226	0.127	1.935	0.252	0.404	0.110
	0.711	0.061	0.132	0.033	0.883	0.084	0.193	0.028
Left hemisphere	2.300	0.276	0.213	0.124	2.003	0.255	0.376	0.110
	0.775	0.063	0.128	0.032	0.869	0.083	0.186	0.027
Right hemisphere	2.288	0.265	0.213	0.124	2.067	0.240	0.395	0.105
	0.727	0.063	0.140	0.035	0.999	0.082	0.197	0.030

	**Persons with aphasia**							
	**Eyes open rest**				**Eyes closed rest**			
	**Delta**	**Theta**	**Alpha**	**Beta**	**Delta**	**Theta**	**Alpha**	**Beta**

Whole brain	1.653	0.361	0.306	0.097	1.526	0.364	0.441	0.076
	0.599	0.138	0.164	0.036	0.510	0.136	0.219	0.027
Clinical	1.697	0.354	0.298	0.098	1.569	0.358	0.434	0.077
	0.650	0.140	0.162	0.036	0.529	0.135	0.215	0.027
Left hemisphere	1.776	0.358	0.267	0.093	1.660	0.365	0.389	0.071
	0.663	0.146	0.134	0.037	0.577	0.147	0.189	0.029
Right hemisphere	1.652	0.314	0.281	0.104	1.579	0.323	0.418	0.084
	0.656	0.130	0.158	0.031	0.590	0.121	0.239	0.027

#### Eyes-Open Rest

Significant differences between controls and PWAs was observed for beta power in the whole brain (*t* = 2.818; *p* = 0.007), clinical (*t* = 2.729; *p* = 0.009), and left hemisphere (*t* = 2.968; *p* = 0.005) montages, and for delta power in the right hemisphere (*t* = 2.97; *p* = 0.005) montage. No significant between-group differences were observed in alpha or theta power, or for delta power in the remaining montages. For all significant comparisons, healthy controls demonstrated greater average power than PWAs. See Table 5 for complete results.

**TABLE 5:** Test statistic, *p*-value, and effect size for between group comparisons during eyes open and eyes closed rest.

	**Eyes open rest**				**Eyes closed rest**			
	**Delta**	**Theta**	**Alpha**	**Beta**	**Delta**	**Theta**	**Alpha**	**Beta**
Whole brain	*t* = *2.663 p* = *0.011 g* = *0.81*	*t* = −*2.188 p* = *0.039 g* = *0.73*	*U* = 313 *Z* = −2.079 *p* = 0.038 η^2^ = 0.10	***t* = 2.818 *p* = 0.007 *g* = 0.89**	*t* = *1.626 p* = *0.112 g* = *0.49*	***t* = −3.122 *p* = 0.004 *g* = 1.01**	*t* = −1.612 *p* = 0.115 *g* = 0.18	***t* = 3.979 *p* ≤ 0.001 *g* = 1.2**
Clinical	*t* = *2.352 p* = *0.024 g* = *0.72*	*t* = −*2.034 p* = *0.053 g* = *0.68*	*U* = 317 *Z* = −2.177 *p* = 0.030 η^2^ = 0.11	***t* = 2.729 *p* = 0.009 *g* = 0.86**	*t* = *1.592 p* = *0.119 g* = *0.48*	***t* = −3.001 *p* = 0.006 *g* = 0.96**	*t* = −0.590 *p* = 0.558 *g* = 0.15	***t* = 3.890 *p* ≤ 0.001 *g* = 1.23**
Left hemisphere	*t* = *2.344 p* = *0.024 g* = *0.72*	*t* = −*2.280 p* = *0.032 g* = *0.77*	*U* = 304 *Z* = −1.859 *p* = 0.063 η^2^ = 0.08	***t* = 2.968 *p* = 0.005 *g* = 0.92**	*t* = *1.480 p* = *0.147 g* = *0.45*	***t* = −2.928 *p* = 0.007 *g* = 0.96**	*t* = −0.023 *p* = 0.820 *g* = 0.07	***t* = 4.529 *p* ≤ 0.001 *g* = 1.45**
Right hemisphere	***t* = 2.970 *p* = 0.005 *g* = 0.91**	*t* = −*1.485 p* = *0.150 g* = *0.49*	*U* = 299 *Z* = −1.736 *p* = 0.082 η^2^ = 0.07	*t* = *1.858 p* = *0.070 g* = *0.58*	*t* = *1.879 p* = *0.067 g* = *0.57*	***t* = −2.673 *p* = 0.011 *g* = 0.82**	*t* = −0.343 *p* = 0.733 *g* = 0.1	***t* = 2.440 *p* = 0.019 *g* = 0.77**

*Comparisons that survived correction for multiple comparisons are bolded. Comparisons that were not statistically significant but showed a medium or large effect size are italicized. For the Eyes Open Alpha comparison only, we report the Z statistic in addition to the Mann–Whitney *U* test statistic to allow a more direct comparison to the Eyes Closed Alpha condition *t*-test statistic results.*

#### Eyes-Closed Rest

Significant differences between controls and PWAs was observed for beta (*t* between 2.44 and 4.529; *p* between 0.019 and <0.001) and theta (*t* between −2.928 and −3.122; *p* between 0.011 and 0.004) power in all montages. No significant between-group differences were observed for alpha or delta power. Compared to PWAs, healthy controls demonstrated greater beta power and lower theta power. See Table 5 for complete results.

### Aim 3 - Functional Relationship

Prior to computing correlations, data were checked for normality and linearity. Due to observations of non-normality in the data, non-parametric Spearman correlations were calculated. Additionally, when plotting data to determine linearity, both the BNT and WAB-R AQ (completed by PWAs only) were determined to be non-linearly related to rsEEG power, so correlations were only computed for the RBANS and Main Concept (MC) scores.

#### Eyes-Open Rest

Moderate to strong negative correlations were observed between MC scores and theta power in the whole brain (rho = −0.5, *p* = 0.029), clinical (rho = −0.49, *p* = 0.033), and left hemisphere (rho = −0.48, *p* = 0.036) montages in PWAs. A moderate positive correlation (rho = 0.49, *p* = 0.039) was also observed between RBANS scores and beta power in the left hemisphere in PWAs. Finally, moderate to strong positive correlations between MC scores and beta power were observed in the whole brain (rho = 0.49, *p* = 0.033), clinical (rho = 0.48, *p* = 0.037), and left hemisphere (rho = 0.58, *p* = 0.009) montages in PWAs. No significant correlations were observed for healthy controls in the eyes-open condition.

#### Eyes-Closed Rest

Strong negative correlations were observed between MC scores and theta power in the whole brain (rho = −0.53, *p* = 0.02), clinical (rho = −0.53, *p* = 0.02), and left hemisphere (rho = −0.55, *p* = 0.014) montages in PWAs. Positive correlations were also observed between MC scores and alpha (rho = 0.59, *p* = 0.008) and beta power (rho = 0.48, *p* = 0.039) in the left hemisphere in PWAs. In healthy controls, strong positive correlations were observed between RBANS scores and theta power in all montages (rho between 0.56 and 0.6, *p* between 0.002 and 0.005).

## Discussion

The results of this study suggest that spectral rsEEG is suitably reliable for use as a repeated measure in both controls and PWAs. Additionally, spectral rsEEG is sensitive to differences between controls and PWAs. Finally, spectral rsEEG measures were also related to behavioral measures of cognition and language.

### Reliability of Spectral rsEEG for Repeated Measurement

To our knowledge, this is the first study to examine the reliability of spectral rsEEG in persons with chronic stroke-induced aphasia – an important step to ensuring these measures are viable for use as neurophysiological biomarkers of treatment response. We selected an approximately 1-month delay between the first and second recording sessions since many aphasia research studies involving a treatment component last approximately 1 month from pre-treatment assessment to post-treatment assessment. Our results demonstrated moderate to good reliability for healthy controls and PWAs in alpha, beta, and theta bands, with relatively poorer reliability in the delta band. Previous research has examined changes in spectral rsEEG over time as a response to treatment (e.g., Rozelle and Budzynski, 1995; Stojanovic et al., 2013). Our results support the use of spectral rsEEG in this manner and strengthen the inferences that can be drawn from such studies. Both groups demonstrated similar spectral rsEEG reliability during the eyes-open and eyes-closed rest conditions, suggesting that either condition may be used for repeated measures, although eyes-closed rest demonstrated slightly better reliability across the board. However, our findings of the overall poor reliability of delta power suggest that researchers should proceed with caution when this is the spectral band of greatest interest, as we expect it could be since it is frequently the focus in acute and sub-acute studies of stroke recovery.

When examining the overall reliability of the various montages, there was not a clear pattern of better reliability in one montage over the others in either condition. However, the left and right hemisphere montage + spectral band combinations were the only ones that demonstrated poor reliability (i.e., left hemisphere delta in the eyes-closed condition for controls and PWAs, right hemisphere delta and theta in the eyes-open condition for controls). This suggests that researchers can have greater confidence in the reliability of results when the montage includes electrodes across both left and right hemisphere, and that additional caution should be taken when examining smaller montages that do not include representation over the whole scalp. Future investigations should continue to explore the impact of montage on reliability, particularly as this sample was comprised mostly of individuals with single hemisphere lesions. However, these results suggest that more clinically focused montages with fewer electrodes should not have significantly worse reliability than the dense electrode arrays more frequently used in research.

When considering the reliability for each group, PWAs demonstrated a pattern of numerically better reliability, especially in the eyes-open condition. It is not immediately clear what is driving this pattern, as our naïve assumption entering into the study was that controls would demonstrate better reliability, given the well-known behavioral variability of PWAs. Although speculative, one possible explanation of this result is that the increased variability in controls is indicative of a system with more flexibility and greater capacity. On the other hand, the decreased variability in PWAs could be indicative of a system that, due to damage, is less able to respond flexibly or has limited capacity. There is some limited preliminary evidence for this in traumatic brain injury during task completion (see Beharelle et al., 2012); however, additional research would be needed to determine if this holds for resting-state activation and in PWAs.

### Utility of Spectral rsEEG to Identify Group Differences

In this study, we observed significant differences in beta (in both conditions) and theta power (in the eyes closed condition only) between controls and PWAs. With respect to theta power, we did not find statistically significant differences between groups in the eyes-open condition, only in the eyes-closed condition. We attribute this to the improved signal-to-noise ratio of the eyes-closed condition, which may have improved statistical power to detect differences, and therefore may have greater clinical and research utility. As expected from research in the acute and sub-acute stages post-stroke, healthy controls demonstrated a pattern of greater power in high frequencies and lower power in lower frequencies than PWAs (but see our discussion of delta power below). These results indicate that some degree of spectral rsEEG slowing observed in the acute and sub-acute stages post-stroke persists into the chronic phase, at least for persons with chronic aphasia. However, our results revealed significant between-group differences in theta power, while much of the previous acute and sub-acute literature has reported significant between-group differences in delta power. While studies examining spectral EEG in the chronic phase have utilized task-based paradigms, thereby limiting the comparisons that can be made to our findings, those investigations did report differences in delta, theta, and beta bands, mostly consistent with our results (Spironelli and Angrilli, 2009; Spironelli et al., 2013; Herron et al., 2014).

Interestingly, while we did observe a single significant difference in delta power between the groups (right hemisphere delta in the eyes open condition), the direction of that difference also ran contrary to previous literature, with healthy controls demonstrating higher delta power than PWAs in the right hemisphere. Indeed, when examining the descriptive statistics of delta power for all montages, healthy controls demonstrated numerically greater delta power across all montages. One possible explanation for this finding is body positioning, which has demonstrated effects on cortical activation (e.g., Spironelli et al., 2016; Thibault and Raz, 2016) - the supine position (laying down) is associated with increased delta activation compared to upright positions. Given that much of the previous research has been conducted in settings where patients are often reclined or laying down in bed, but our study only included participants who were sitting upright in a chair, it is possible that the positioning differences between this study and previous research may at least partially explain this dissimilar finding.

Additionally, since most studies have examined persons post-stroke in the acute and sub-acute, rather than chronic phase, it is possible that the slowing of spectral rsEEG resolves to a certain extent. This is consistent with Hensel et al. (2004) who conducted a longitudinal study of post-stroke recovery and reported a gradual increase in the frequency of spectral rsEEG over the course of 2 years. Another alternative explanation lies in the sample characteristics of participants in our study (e.g., specifically investigating aphasia, including persons with multiple strokes, mild depression or anxiety, and a realistic range of control performance). It is also consistent with patterns observed via fMRI where the locus of activation during a language tasks shifts from the right hemisphere in the acute stage back toward the left hemisphere in the sub-acute and chronic stages (Heiss et al., 1999; Saur et al., 2006). Still, while our more lenient inclusion/exclusion criteria may have increased the variability of the sample, the ability to directly compare our results to the typical clinical population served by practicing speech-language pathologists, and a more realistic cross-section of the population, compensates for loss of specificity.

### Relationship of Spectral rsEEG Power With Behavior

We investigated the correlation between spectral rsEEG measures and performance on behavioral tasks, as the relationship between spectral rsEEG and behavioral performance has been frequently reported in the acute and sub-acute spectral rsEEG literature. Our results provide an important assurance that in addition to being reliable over time, the differences in spectral rsEEG observed between PWAs and controls relate to functionally relevant behaviors. Results also support the notion of a continued relationship between spectral rsEEG and behavioral function in the chronic phase of stroke recovery. Of greatest interest, we found that the main concept (MC) composite score, a measure of discourse informativeness, was positively correlated with power in high frequency bands, and negatively correlated with power in low frequency bands. These findings indicate that spectral rsEEG slowing observed in PWAs in the chronic stage is pathological and suggests a possible avenue for directly altering brain activation to improve behavioral function. However, additional research is needed to fully elucidate the relationship between spectral rsEEG and the behaviors included here. While it may be tempting to try to interpret these results in the context of spectral task-based EEG findings (e.g., cortical inhibition and/or activation variously associated with the spectral bands Engel and Fries, 2010; Palva and Palva, 2011; Cavanagh and Frank, 2014; Cavanagh, 2015; Antzoulatos and Miller, 2016; Richter et al., 2017), these associations do not necessarily hold for resting-state measurements. Therefore, cautious interpretation of the mechanistic processes inferred by spectral bands at rest is recommended.

Our finding of a strong positive correlation between theta power and RBANS scores in healthy controls was relatively unexpected. This result suggests there might be a range of resting theta power that is functionally appropriate, and within this range, increased theta is generally associated with better cognition. However, once this range of theta values is exceeded (as in persons with stroke) higher resting theta power may be functionally maladaptive. This interpretation is supported by the fact that higher resting state theta power is associated with better cognitive function in healthy aging (Cummins and Finnigan, 2007) and when comparing young adults to older adults (Finnigan and Robertson, 2011).

These results also highlight the importance of the behavioral measures selected to quantify functional abilities. While the WAB-R is a standardized and norm-referenced assessment, it demonstrated a ceiling effect in this sample, as it often does in aphasia research and practice. The WAB-R is scored out of 100 points, and the cut-off score to distinguish between PWAs and those without aphasia is 93.8. This means that the entire range of “normal” or “not aphasic” performance is constrained to less than seven points in comparison to the range of 93.8 points to quantify aphasic performance. Further, it is well-known that this score is not sensitive to mild aphasic deficits that may be most prominent in connected speech, which it does not adequately assess. Similarly, the BNT is scored out of 60 points, and most healthy controls correctly name 53–60 items (depending on age and education; Tombaugh and Hubiey, 1997). While naming is the hallmark impairment of aphasia, confrontation naming tasks such as the BNT may not adequately represent difficulties in connected speech (e.g., Richardson et al., 2018). In contrast, the MC composite score has demonstrated sufficient sensitivity to describe both impaired and control discourse across a wide range of performance (Dalton and Richardson, 2019; Richardson et al., 2021). These results further amplify calls to utilize discourse measures as the primary outcome for aphasia research studies since discourse performance is a better index of the functional communication outcomes desired by PWAs (Brady et al., 2016). To our knowledge, this is the first study to relate spectral rsEEG to discourse production abilities in PWAs.

### Limitations and Future Directions

Future research should more directly examine the effect of lesion location in PWAs as research in individuals in the chronic stage post-stroke suggests this may have an effect on spectral rsEEG (e.g., Park et al., 2016). Because this study included PWAs with fluent and non-fluent aphasia, lesion locations included both posterior and anterior left hemisphere perisylvian regions (see Supplementary Table 1), as well as subcortical areas and right hemisphere (for individuals with mixed hemisphericity and/or multiple strokes). A more nuanced understanding of the results with improved localization of the spectral rsEEG sources and region of interest analyses with functional networks may be possible when EEG is paired with other neuroimaging modalities, such as MRI or MEG. Additional avenues for future research include examining changes in spectral rsEEG before and after speech-language therapy to determine the sensitivity and predictive capacity of spectral rsEEG and investigating the reliability of functional behavioral measures, such as the discourse measures included in this study, to identify sources of non-informative variability that can be minimized in the future.

Improvements in reporting methodology and basic descriptive statistical information regarding data are needed. For example, we excluded individuals with clinically identified depression and anxiety, but allowed individuals without a clinical diagnosis who reported experiencing mild symptoms of depression and/or anxiety. This decision was based on the reportedly high prevalence of depression and/or anxiety in PWAs (e.g., Hilari et al., 2012; Ayerbe et al., 2013; Døli et al., 2017; Morris et al., 2017) as well as precedent set by previous foundational spectral EEG studies in chronic stroke with and without aphasia. Most studies of spectral EEG in individuals’ post-stroke with or without aphasia do not mention mental health disorders as an exclusion criterion, so it is unclear whether individuals in their sample experience these difficulties. Those studies that do report an exclusion criterion for mental health issues range from excluding only individuals with refractory (or treatment-resistant) depression (Carrick et al., 2016), to excluding individuals who are taking specific classes of medications (such as benzodiazepines or tricyclic antidepressants, e.g., Finnigan et al., 2007), to excluding based on known (Nicolo et al., 2015; Song et al., 2015; Finnigan et al., 2016; Gorišek et al., 2016), major active (Wu et al., 2015), major pre-morbid (Leon-Carrion et al., 2009), and/or uncontrolled (Herron et al., 2014) psychiatric illness. In this study, prospective data regarding the types or severity of depression and anxiety symptoms were not collected since mental health concerns were asked about during pre-enrollment screening only. The lack of this data limits the interpretation of the results presented here, as it is possible that between group differences, or lack of differences, were driven by altered spectral rsEEG secondary to depression and/or anxiety. In the future, more clearly defining inclusion/exclusion criteria (e.g., what is the difference between “major active” and “known” psychiatric illness?), prospectively collecting depression and anxiety symptoms and severity in both clinical and healthy control participants using measures validated for those populations, and clear reporting of these characteristics may yield even greater insights into brain function.

By providing more detailed methodology and data reporting, we can increase the confidence in reported results and strengthen the inferences that can be drawn from published findings. Ideally, a database, such as those already established for healthy controls and some populations with disorders (e.g., Brain Research and Integrative Neuroscience Network^1^ ; Patient Repository for EEG Data + Computational Tools^2^) would be populated with data from PWAs to allow for sharing of data and use of big data analytics that are currently unavailable. The current lack of such data in existing repositories highlights the need for continued research specific to PWAs. Much of the stroke literature actively excludes PWAs from participation, typically due to concerns regarding language abilities, informed consent, and the ability to complete language-based study activities (Thorne and Paterson, 2000; Townend et al., 2007; Dalemans et al., 2009; Brady et al., 2013). Unfortunately, even that research which does not exclude PWAs often neglects to report their data as a separate subset of the participants. Since there is sufficient evidence in the literature that PWAs are impacted differently, and more severely, than individuals with other types of post-stroke deficits (e.g., Hilari, 2011; Brady et al., 2013; Hilari and Northcott, 2017; Pike et al., 2017; Simmons-Mackie, 2018), the inclusion of, and separate reporting of data for, PWAs is warranted.

## Conclusion

Within the field of stroke rehabilitation, and especially within the field of aphasiology, there is great need for improved individualization and optimization of rehabilitation. One of the primary limiting factors in achieving this goal is the lack of sensitive measures that can predict treatment response. Simply looking at an individual’s behavioral profile or examining structural or functional MRI records has proven insufficient to determine the treatment course that will lead to greatest functional recovery for that individual. This is especially critical for adults engaging in rehabilitation, since private insurance companies may impose annual caps on total therapy hours or dollars, or limit access to therapy for individuals with mild or latent, but functionally debilitating or limiting, deficits. By improving individualization of treatment and thereby maximizing outcomes, PWAs will be more likely to experience meaningful improvements in everyday living.

Resting-state investigations, both rsEEG and rsfMRI, hold great advantages for the inclusion of PWAs (or others with specific post-stroke deficits that alter behavioral task performance) in research studies. Resting-state paradigms allow investigations of functional networks without the confounds of response accuracy, variable cognitive load across participants, and the differential recruitment of brain areas in response to cognitive load. In this study, speech-language pathologists and speech-language pathology master’s students with little to no background in EEG were able to collect high quality data, regardless of the severity of participant deficits, following provision of a detailed study procedures manual, several training (including practice) sessions, and minimal supervision during initial participant sessions. Spectral rsEEG, especially when recorded in an eyes-closed condition, shows potential as a means of improving diagnostic sensitivity, tracking outcomes, and individualizing therapy for maximum benefit given its reliability, persistent changes after stroke, and correlations with behavioral function.

## Data Availability Statement

The raw data supporting the conclusions of this article will be made available by the authors, without undue reservation.

## Ethics Statement

The studies involving human participants were reviewed and approved by Institutional Review Board – Human Research Review Committee University of New Mexico Health Sciences. The patients/participants provided their written informed consent to participate in this study.

## Author Contributions

SD contributed to all aspects of the research process including study conception and design, data collection, data analysis, and manuscript drafting. JC contributed to study conception and design, data analysis, and manuscript revisions. JR contributed to study conception and design, data collection, and manuscript revisions. All authors contributed to the article and approved the submitted version.

## Conflict of Interest

The authors declare that the research was conducted in the absence of any commercial or financial relationships that could be construed as a potential conflict of interest.

## Footnotes

1 http://www.brainnet.net

2 http://predict.cs.unm.edu

## References

[B1] Aickin M. Gensler H. (1996). Adjusting for multiple testing when reporting research results: the Bonferroni vs Holm methods. *Am. J. Public Health* 86 726–728. 10.2105/ajph.86.5.726 8629727 PMC1380484

[B2] Antzoulatos E. G. Miller E. K. (2016). Synchronous beta rhythms of frontoparietal networks support only behaviorally relevant representations. *eLife* 5:e17822. 10.7554/eLife.17822 PMC5148609 27841747

[B3] Ayerbe L. Ayis S. Wolfe C. D. Rudd A. G. (2013). Natural history, predictors and outcomes of depression after stroke: systematic review and meta-analysis. *Br. J. Psychiatry* 202 14–21. 10.1192/bjp.bp.111.107664 23284148

[B4] Barry R. J. De Blasio F. M. (2017). EEG differences between eyes-closed and eyes-open resting remain in healthy ageing. *Biol. Psychol.* 129 293–304. 10.1016/j.biopsycho.2017.09.010 28943465

[B5] Barry R. J. Clarke A. R. Johnstone S. J. Magee C. A. Rushby J. A. (2007). EEG differences between eyes-closed and eyes-open resting conditions. *Clin. Neurophysiol.* 118 2765–2773. 10.1016/j.clinph.2007.07.028 17911042

[B6] Beharelle A. R. Kovačević N. McIntosh A. R. Levine B. (2012). Brain signal variability relates to stability of behavior after recovery from diffuse brain injury. *Neuroimage* 60 1528–1537. 10.1016/j.neuroimage.2012.01.037 22261371 PMC3303989

[B7] Bolduc C. Daoust A. M. Limoges É Braun C. M. Godbout R. (2003). Hemispheric lateralization of the EEG during wakefulness and REM sleep in young healthy adults. *Brain Cogn.* 53 193–196. 10.1016/s0278-2626(03)00108-8 14607146

[B8] Boneau C. A. (1960). The effects of violations of assumptions underlying the t test. *Psychol. Bull.* 57 49–64. 10.1037/h0041412 13802482

[B9] Borghini G. Aricò P. Flumeri G. D. Cartocci G. Colosimo A. Bonelli S. (2017). EEG-Based cognitive control behaviour assessment: an ecological study with professional air traffic controllers. *Sci. Rep.* 7:547. 10.1038/s41598-017-00633-7 28373684 PMC5428823

[B10] Bradley J. V. (1982). The insidious L-shaped distribution. *Bull. Psycho. Soc.* 20 85–88. 10.3758/bf03330089

[B11] Brady M. C. Fredrick A. Williams B. (2013). People with aphasia: capacity to consent, research participation and intervention inequalities. *Int. J. Stroke* 8 193–196. 10.1111/j.1747-4949.2012.00900.x 23130972

[B12] Brady M. C. Kelly H. Godwin J. Enderby P. Campbell P. (2016). Speech and language therapy for aphasia following stroke. *Cochrane Database Syst. Rev.* 6 1–308. 10.1002/14651858.cd000425.pub4 27245310 PMC8078645

[B13] Brookshire R. H. Nicholas L. E. (1997). *Discourse Comprehension Test: Test Manual.* Minneapolis, MN: BRK Publishers.

[B14] Carrick F. R. Oggero E. Pagnacco G. Wright C. H. Machado C. Estrada G. (2016). Eye-movement training results in changes in qEEG and NIH stroke scale in subjects suffering from acute middle cerebral artery ischemic stroke: a randomized control trial. *Front. Neurol.* 7:3. 10.3389/fneur.2016.00003 PMC4722822 26834698

[B15] Cavanagh J. F. (2015). Cortical delta activity reflects reward prediction error and related behavioral adjustments, but at different times. *Neuroimage* 110 205–216. 10.1016/j.neuroimage.2015.02.007 25676913

[B16] Cavanagh J. F. Frank M. J. (2014). Frontal theta as a mechanism for cognitive control. *Trends Cogn. Sci.* 18 414–421. 10.1016/j.tics.2014.04.012 24835663 PMC4112145

[B17] Chatrian G. E. Lettich E. Nelson P. L. (1985). Ten percent electrode system for topographic studies of spontaneous and evoked EEG activities. *Am. J. EEG Technol.* 25 83–92. 10.1080/00029238.1985.11080163

[B18] Cohen J. (1988). *Statistical Power Analysis for the Behavioral Sciences.* Hillsdale, NJ: L. Erlbaum Associates.

[B19] Corsi-Cabrera M. Solís-Ortiz S. Guevara M. (1997). Stability of EEG inter-and intrahemispheric correlation in women. *Electroencephalogr. Clin. Neurophysiol.* 102 248–255. 10.1016/s0013-4694(96)95179-6 9129580

[B20] Cummins T. D. Finnigan S. (2007). Theta power is reduced in healthy cognitive aging. *Int. J. Psychophysiol.* 66 10–17. 10.1016/j.ijpsycho.2007.05.008 17582632

[B21] Cuspineda E. Machado C. Aubert E. Galán L. Liopis F. Avila Y. (2003). Predicting outcome in acute stroke: a comparison between QEEG and the Canadian Neurological Scale. *Clin. Electroencephalogr.* 34 1–4. 10.1177/155005940303400104 12515444

[B22] Dabul B. (2000). *Apraxia Battery for Adults*, 2nd Edn. Austin, TX: Pro-Ed.

[B23] Dalemans R. Wade D. T. Heuvel W. J. Witte L. P. (2009). Facilitating the participation of people with aphasia in research: a description of strategies. *Clin. Rehabil.* 23 948–959. 10.1177/0269215509337197 19570814

[B24] Dalton S. G. Hubbard H. I. Richardson J. D. (2020). Moving toward non-transcription based discourse analysis in stable and progressive aphasia. *Semin. Speech Lang.* 41 032–044. 10.1055/s-0039-3400990 31869847 PMC11363584

[B25] Dalton S. G. Richardson J. D. (2019). A large-scale comparison of main concept production between persons with aphasia and persons without brain injury. *Am. J. Speech Lang. Pathol.* 28 293–320. 10.1044/2018_ajslp-17-0166 31072179 PMC6437704

[B26] de Vos C. C. van Maarseveen S. M. Brouwers P. J. van Putten M. J. (2008). Continuous EEG monitoring during thrombolysis in acute hemispheric stroke patients using the brain symmetry index. *J. Clin. Neurophysiol.* 25 77–82. 10.1097/wnp.0b013e31816ef725 18340268

[B27] Døli H. Helland T. Andersen Helland W. (2017). Self-reported symptoms of anxiety and depression in chronic stroke patients with and without aphasia. *Aphasiology* 31 1392–1409. 10.1080/02687038.2017.1280595

[B28] Dubovik S. Ptak R. Aboulafia T. Magnin C. Gillabert N. Allet L. (2013). EEG alpha band synchrony predicts cognitive and motor performance in patients with ischemic stroke. *Behav. Neurol.* 26 187–189. 10.1155/2013/109764 22713421 PMC5214220

[B29] Engel A. K. Fries P. (2010). Beta-band oscillations—signalling the status quo? *Curr. Opin. Neurobiol.* 20 156–165. 10.1016/j.conb.2010.02.015 20359884

[B30] Finnigan S. van Putten M. J. (2013). EEG in ischaemic stroke: quantitative EEG can uniquely inform (sub-)acute prognoses and clinical management. *Clin. Neurophysiol.* 124 10–19. 10.1016/j.clinph.2012.07.003 22858178

[B31] Finnigan S. Robertson I. H. (2011). Resting EEG theta power correlates with cognitive performance in healthy older adults. *Psychophysiology* 48 1083–1087. 10.1111/j.1469-8986.2010.01173.x 21729101

[B32] Finnigan S. Wong A. Read S. (2016). Defining abnormal slow EEG activity in acute ischaemic stroke: delta/alpha ratio as an optimal QEEG index. *Clin. Neurophysiol.* 127 1452–1459. 10.1016/j.clinph.2015.07.014 26251106

[B33] Finnigan S. P. Walsh M. Rose S. E. Chalk J. B. (2007). Quantitative EEG indices of sub-acute ischaemic stroke correlate with clinical outcomes. *Clin. Neurophysiol.* 118 2525–2532. 10.1016/j.clinph.2007.07.021 17889600

[B34] French C. C. Beaumont J. G. (1984). A critical review of EEG coherence studies of hemisphere function. *Int. J. Psychophysiol.* 1 241–254. 10.1016/0167-8760(84)90044-8 6394561

[B35] Fromm D. Forbes M. Holland A. Dalton S. G. Richardson J. Macwhinney B. (2017). Discourse characteristics in aphasia beyond the western aphasia battery cutoff. *Am. J. Speech Lang. Pathol.* 26 762–768. 10.1044/2016_ajslp-16-0071 28505222 PMC5829792

[B36] Fromm D. Forbes M. Holland A. Macwhinney B. (2020). Using AphasiaBank for discourse assessment. *Semin. Speech Lang.* 41 010–019. 10.1055/s-0039-3399499 31869845 PMC8915263

[B37] Gorišek V. R. Isoski V. Z. Belič A. Manouilidou C. Koritnik B. Bon J. (2016). Beyond aphasia: altered EEG connectivity in Broca’s patients during working memory task. *Brain Lang.* 163 10–21. 10.1016/j.bandl.2016.08.003 27631161

[B38] Gow D. W. Jr. Ahlfors S. P. (2017). Tracking reorganization of large-scale effective connectivity in aphasia following right hemisphere stroke. *Brain Lang.* 170 12–17. 10.1016/j.bandl.2017.03.003 28364641 PMC5472378

[B39] Gudmundsson S. Runarsson T. P. Sigurdsson S. Eiriksdottir G. Johnsen K. (2007). Reliability of quantitative EEG features. *Clin. Neurophysiol.* 118 2162–2171. 10.1016/j.clinph.2007.06.018 17765604

[B40] Hamming R. W. (1998). *Digital Filters.* Mineola, NY: Dover Publications, 252.

[B41] Heiss W. D. Kessler J. Thiel A. Ghaemi M. Karbe H. (1999). Differential capacity of left and right hemispheric areas for compensation of poststroke aphasia. *Ann. Neurol.* 45 430–438. 10.1002/1531-8249(199904)45:4<430::aid-ana3>3.0.co;2-p 10211466

[B42] Hensel S. Rockstroh B. Berg P. Elbert T. Schönle P. W. (2004). Left-hemispheric abnormal EEG activity in relation to impairment and recovery in aphasic patients. *Psychophysiology* 41 394–400. 10.1111/j.1469-8986.2004.00164x 15102124

[B43] Herron K. Dijk D. Dean P. Seiss E. Sterr A. (2014). Quantitative electroencephalography and behavioural correlates of daytime sleepiness in chronic stroke. *BioMed. Res. Int.* 2014 1–11. 10.1155/2014/794086 24883327 PMC4032711

[B44] Hilari K. (2011). The impact of stroke: are people with aphasia different to those without? *Disability Rehabil.* 33 211–218. 10.3109/09638288.2010.508829 20712416

[B45] Hilari K. Needle J. J. Harrison K. L. (2012). What are the important factors in health-related quality of life for people with aphasia? A systematic review. *Arch. Phys. Med. Rehabil.* 93 S86–S95. 22119074 10.1016/j.apmr.2011.05.028

[B46] Hilari K. Northcott S. (2017). “Struggling to stay connected”: comparing the social relationships of healthy older people and people with stroke and aphasia. *Aphasiology* 31 674–687. 10.1080/02687038.2016.1218436

[B47] Holm S. (1979). A simple sequentially rejective multiple test procedure. *Scand. J. Stat.* 6 65–70.

[B48] Ioannidis J. P. Bossuyt P. M. (2017). Waste, leaks, and failures in the biomarker pipeline. *Clin. Chem.* 63 963–972. 10.1373/clinchem.2016.254649 28270433

[B49] Kaplan E. Goodglass H. Weintraub S. (2001). *Boston Naming Test.* Austin, TX: Pro-ed.

[B50] Kertesz A. (2006). *Western Aphasia Battery – Revised.* San Antonio, TX: Pearson.

[B51] Klingbeil J. Wawrzyniak M. Stockert A. Saur D. (2019). Resting-state functional connectivity: an emerging method for the study of language networks in post-stroke aphasia. *Brain Cogn.* 131 22–33. 10.1016/j.bandc.2017.08.005 28865994

[B52] Kondacs A. Szabó M. (1999). Long-term intra-individual variability of the background EEG in normals. *Clin. Neurophysiol.* 110 1708–1716. 10.1016/s1388-2457(99)00122-4 10574286

[B53] Koo T. K. Li M. Y. (2016). A guideline of selecting and reporting intraclass correlation coefficients for reliability research. *J. Chiropractic Med.* 15 155–163. 10.1016/j.jcm.2016.02.012 27330520 PMC4913118

[B54] Learmonth G. Benwell C. S. Thut G. Harvey M. (2017). Age-related reduction of hemispheric lateralisation for spatial attention: an EEG study. *Neuroimage* 153 139–151. 10.1016/j.neuroimage.2017.03.050 28343987

[B55] Leon-Carrion J. Martin-Rodriguez J. F. Damas-Lopez J. y Martin J. M. B. Dominguez-Morales M. R. (2009). Delta–alpha ratio correlates with level of recovery after neurorehabilitation in patients with acquired brain injury. *Clin. Neurophysiol.* 120 1039–1045. 10.1016/j.clinph.2009.01.021 19398371

[B56] Logan G. D. (1985). Executive control of thought and action. *Acta Psychol.* 60 193–210. 10.1016/0001-6918(85)90055-1

[B57] Makeig S. Bell A. J. Jung T. P. Sejnowski T. J. (1996). Independent component analysis of electroencephalographic data. *Adv. Neural Information Processing Syst.* 8 145–151.

[B58] Marian V. Blumenfeld H. K. Kaushanskaya M. (2007). The language experience and proficiency questionnaire (LEAP-Q): assessing language profiles in bilinguals and multilinguals. *J. Speech Lang. Hear. Res.* 50 940–967. 10.1044/1092-4388(2007/067) 17675598

[B59] McGraw K. O. Wong S. P. (1996). Forming inferences about some intraclass correlation coefficients. *Psychol. Methods* 1:30. 10.1037/1082-989x.1.1.30

[B60] Morris R. Eccles A. Ryan B. Kneebone I. I. (2017). Prevalence of anxiety in people with aphasia after stroke. *Aphasiology* 31 1410–1415. 10.1080/02687038.2017.1304633

[B61] Nicholas L. E. Brookshire R. H. (1995). Presence, completeness, and accuracy of main concepts in the connected speech of non-brain-damaged adults and adults with aphasia. *J. Speech Lang. Hear. Res.* 38 145–156. 10.1044/jshr.3801.145 7731205

[B62] Nicolo P. Rizk S. Magnin C. Pietro M. D. Schnider A. Guggisberg A. G. (2015). Coherent neural oscillations predict future motor and language improvement after stroke. *Brain* 138 3048–3060. 10.1093/brain/awv200 26163304

[B63] Nolfe G. Cobianchi A. Mossuto-Agatiello L. Giaquinto S. (2006). The role of P300 in the recovery of post-stroke global aphasia. *Eur. J. Neurol.* 13 377–384. 10.1111/j.1468-1331.2006.01237.x 16643316

[B64] Oken B. Chiappa K. (1988). Short-term variability in EEG frequency analysis. *Electroencephalogr. Clin. Neurophysiol.* 69 191–198. 10.1016/0013-4694(88)90128-9 2450000

[B65] Oppenheim A. Schafer R. W. Buck J. R. (1999). *Discrete-Time Signal Processing*, 2nd Edn. Upper Saddle River, NJ: Prentice Hall.

[B66] Othman E. A. Yusoff A. N. Mohamad M. Abdul Manan H. Abd Hamid A. I. Giampietro V. (2020). Hemispheric lateralization of auditory working memory regions during stochastic resonance: an fMRI study. *J. Magnetic Resonance Imaging* 51 1821–1828. 10.1002/jmri.27016 31794119

[B67] Palva S. Palva J. M. (2011). Functional roles of alpha-band phase synchronization in local and large-scale cortical networks. *Front. Psychol.* 2:204. 10.3389/fpsyg.2011.00204 PMC3166799 21922012

[B68] Park W. Kwon G. H. Kim Y. Lee J. Kim L. (2016). EEG response varies with lesion location in patients with chronic stroke. *J. NeuroEngineering Rehabil.* 13:21. 10.1186/s12984-016-0120-2 26935230 PMC4776402

[B69] Petrovic J. Milosevic V. Zivkovic M. Stojanov D. Milojkovic O. Kalauzi A. (2017). Slower EEG alpha generation, synchronization and “flow”—possible biomarkers of cognitive impairment and neuropathology of minor stroke. *PeerJ* 5:e3839. 10.7717/peerj.3839 28970969 PMC5623310

[B70] Piai V. Meyer L. Dronkers N. F. Knight R. T. (2017). Neuroplasticity of language in left hemisphere stroke: evidence linking subsecond electrophysiology and structural connections. *Hum. Brain Mapp.* 38 3151–3162. 10.1002/hbm.23581 28345282 PMC5610921

[B71] Pike C. Kritzinger A. Pillay B. (2017). Social participation in working-age adults with aphasia: an updated systematic review. *Top. Stroke Rehabil.* 24 627–639. 10.1080/10749357.2017.1366012 28851257

[B72] Posten H. O. (1978). The robustness of the two-sample t-test over the Pearson system. *J. Stat. Comput. Simulation* 6 295–311. 10.1080/00949657808810197

[B73] Price C. J. Crinion J. Friston K. J. (2006). Design and analysis of fMRI studies with neurologically impaired patients. *J. Magn. Reson. Imaging* 23 816–826. 10.1002/jmri.20580 16649208

[B74] Randolph C. (1998). *Repeatable Battery for the Assessment of Neuropsychological Status (RBANS).* San Antonio, TX: Psychological Corporation.

[B75] Richardson J. D. Dalton S. G. (2016). Main concepts for three different discourse tasks in a large non-clinical sample. *Aphasiology* 30 45–73. 10.1080/02687038.2015.1057891 PMC11864783 40012631

[B76] Richardson J. D. Dalton S. G. (2020). Main concepts for two picture description tasks: an addition to Richardson and Dalton, 2016. *Aphasiology* 34 119–136. 10.1080/02687038.2018.1561417 32952259 PMC7500506

[B77] Richardson J. D. Dalton S. G. Greenslade K. J. Jacks A. Haley K. L. Adams J. (2021). Main concept, sequencing, and story grammar analyses of cinderella narratives in a large sample of persons with aphasia. *Brain Sci.* 11:110. 10.3390/brainsci11010110 33467602 PMC7830981

[B78] Richardson J. D. Hudspeth Dalton S. G. Fromm D. Forbes M. Holland A. MacWhinney B. (2018). The relationship between confrontation naming and story gist production in aphasia. *Am. J. Speech Lang. Pathol.* 27 406–422. 10.1044/2017_ajslp-16-0211 29497752 PMC6111489

[B79] Richter C. G. Thompson W. H. Bosman C. A. Fries P. (2017). Top-down beta enhances bottom-up gamma. *J. Neurosci.* 37 6698–6711. 10.1523/jneurosci.3771-16.2017 28592697 PMC5508256

[B80] Ross E. D. Monnot M. (2011). Affective prosody: what do comprehension errors tell us about hemispheric lateralization of emotions, sex and aging effects, and the role of cognitive appraisal. *Neuropsychologia* 49 866–877. 10.1016/j.neuropsychologia.2010.12.024 21182850

[B81] Rozelle G. R. Budzynski T. H. (1995). Neurotherapy for stroke rehabilitation: a single case study. *Biofeedback Self-Regulation* 20 211–228. 10.1007/bf01474514 7495916

[B82] Ruxton G. D. (2006). The unequal variance t-test is an underused alternative to Student’s t-test and the Mann–Whitney U test. *Behav. Ecol.* 17 688–690. 10.1093/beheco/ark016 27193460

[B83] Salinsky M. Oken B. Morehead L. (1991). Test-retest reliability in EEG frequency analysis. *Electroencephalogr. Clin. Neurophysiol.* 79 382–392. 10.1016/0013-4694(91)90203-g 1718711

[B84] Sandberg C. W. (2017). Hypoconnectivity of resting-state networks in persons with aphasia compared with healthy age-matched adults. *Front. Hum. Neurosci.* 11:91. 10.3389/fnhum.2017.00091 PMC5329062 28293185

[B85] Saur D. Lange R. Baumgaertner A. Schraknepper V. Willmes K. Rijntjes M. (2006). Dynamics of language reorganization after stroke. *Brain* 129 1371–1384. 10.1093/brain/awl090 16638796

[B86] Schleiger E. Sheikh N. Rowland T. Wong A. Read S. Finnigan S. (2014). Frontal EEG delta/alpha ratio and screening for post-stroke cognitive deficits: the power of four electrodes. *Int. J. Psychophysiol.* 94 19–24. 10.1016/j.ijpsycho.2014.06.012 24971913

[B87] Schmider E. Ziegler M. Danay E. Beyer L. Bühner M. (2010). Is it really robust? *Methodology* 6 147–151. 10.1027/1614-2241/a000016

[B88] Sheorajpanday R. V. Nagels G. Weeren A. J. Putten M. J. Deyn P. P. (2009). Reproducibility and clinical relevance of quantitative EEG parameters in cerebral ischemia: a basic approach. *Clin. Neurophysiol.* 120 845–855. 10.1016/j.clinph.2009.02.171 19375386

[B89] Shrout P. E. Fleiss J. L. (1979). Intraclass correlations: uses in assessing rater reliability. *Psychol. Bull.* 86:420. 10.1037/0033-2909.86.2.420 18839484

[B90] Simmons-Mackie N. (2018). *The State of Aphasia in North America.* Moorestown, NJ: Aphasia Access.

[B91] Song Y. Zang D. W. Jin Y. Y. Wang Z. J. Ni H. Y. Yin J. Z. (2015). Background rhythm frequency and theta power of quantitative eeg analysis: predictive biomarkers for cognitive impairment post–cerebral infarcts. *Clin. Electroencephalogr. Neurosci.* 46 142–146. 10.1177/1550059413517492 24699438

[B92] Spironelli C. Angrilli A. (2009). EEG delta band as a marker of brain damage in aphasic patients after recovery of language. *Neuropsychologia* 47 988–994. 10.1016/j.neuropsychologia.2008.10.019 19027029

[B93] Spironelli C. Busenello J. Angrilli A. (2016). Supine posture inhibits cortical activity: evidence from Delta and Alpha EEG bands. *Neuropsychologia* 89 125–131. 10.1016/j.neuropsychologia.2016.06.015 27312745

[B94] Spironelli C. Manfredi M. Angrilli A. (2013). Beta EEG band: a measure of functional brain damage and language reorganization in aphasic patients after recovery. *Cortex* 49 2650–2660. 10.1016/j.cortex.2013.05.003 23810123

[B95] Stojanovic B. Djurasic L. Jovic S. Paspalj D. (2013). EEG study of visual reactivity in aphasic patients. *Acta Chirurgica Iugoslavica* 60 45–56. 10.2298/aci1303045s 24669580

[B96] Suarez-Revelo J. X. Ochoa-Gomez J. F. Duque-Grajales J. Montoya-Betancur A. Sanchez-Lopez S. (2015). “Test-retest reliability in electroencephalographic recordings,” in *Proceedings of the 2015 20th Symposium on Signal Processing, Images and Computer Vision (STSIVA)* (Bogota). 10.1109/stsiva.2015.7330412

[B97] Szaflarski J. P. Holland S. K. Schmithorst V. J. Byars A. W. (2006). fMRI study of language lateralization in children and adults. *Hum. Brain Mapp.* 27 202–212. 10.1002/hbm.20177 16035047 PMC1464420

[B98] Thibault R. T. Raz A. (2016). Imaging posture veils neural signals. *Front. Hum. Neurosci.* 10:520. 10.3389/fnhum.2016.00520 27818629 PMC5073137

[B99] Thorne S. E. Paterson B. L. (2000). Two decades of insider research: what we know and don’t know about chronic illness experience. *Annu. Rev. Nurs. Res.* 18 3–25. 10.1891/0739-6686.18.1.3 10918930

[B100] Tombaugh T. N. Hubiey A. M. (1997). The 60-item Boston naming test: norms for cognitively intact adults aged 25 to 88 years. *J. Clin. Exp. Neuropsychol.* 19 922–932. 10.1080/01688639708403773 9524887

[B101] Townend E. Brady M. Mclaughlan K. (2007). Exclusion and inclusion criteria for people with aphasia in studies of depression after stroke: a systematic review and future recommendations. *Neuroepidemiology* 29 1–17. 10.1159/000108913 17898519

[B102] Veldsman M. Cumming T. Brodtmann A. (2014). Beyond BOLD: optimizing functional imaging in stroke populations. *Hum. Brain Mapp.* 36 1620–1636. 10.1002/hbm.22711 25469481 PMC6869358

[B103] Wechsler D. Coalson D. L. Raiford S. E. (2008). *WAIS-IV: Wechsler Adult Intelligence Scale.* San Antonio, TX: Pearson.

[B104] Wilson S. M. Eriksson D. K. Yen M. Demarco A. T. Schneck S. M. Lucanie J. M. (2019). Language mapping in aphasia. *J. Speech Lang. Hear. Res.* 62 3937–3946. 31756153 10.1044/2019_JSLHR-L-RSNP-19-0031 PMC7203526

[B105] Wu J. Quinlan E. B. Dodakian L. Mckenzie A. Kathuria N. Zhou R. J. (2015). Connectivity measures are robust biomarkers of cortical function and plasticity after stroke. *Brain* 138 2359–2369. 10.1093/brain/awv156 26070983 PMC4840951

