# Supplementary material for: Spectral Resting-State EEG (rsEEG) in Chronic Aphasia Is Reliable, Sensitive, and Correlates With Functional Behavior
Source: Front Hum Neurosci. 2021 Mar 17;15:624660. doi: 10.3389/fnhum.2021.624660 (PMC8010195; doi:10.3389/fnhum.2021.624660)
Supplement: Supplementary file 1 [file Table_1.pdf]

Supplementary Material

Supplemental Table 1. Individual demographic information for PWA participants.

Par	Sex	Age	Edu	# of Strokes	TPO	Lesion Location(s)	Aphasia Subtype	Cognitive Deficit	Sensorimotor Deficit
**1**	F	56	18	1	183	Left temporal	Not Aphasic by WAB	Yes	Yes
**2**	M	55	12	3	26	Left temporal; right frontal, occipital, parietal, temporal	Anomic Aphasia	No	Yes
**3**	M	48	16	2	15	Left frontal, insula, parietal, temporal; left basal ganglia	Not Aphasic by WAB	No	No
**4**	M	55	14	1	36	Left frontal, insula, parietal, temporal; left basal ganglia	Transcortical Motor Aphasia	Yes	No
**5**	M	53	14	1	92	Left frontal, insula, parietal, temporal; left basal ganglia; right frontal, parietal	Broca's Aphasia	Yes	Yes
**6**	M	53	20	1	5	Left frontal, insula, temporal; left basal ganglia, hippocampus, thalamus	Not Aphasic by WAB	No	Yes
**7**	M	69	14	1	35	Left frontal, insula	Anomic Aphasia	No	Yes
**8**	F	33	14	2	38	Left frontal; left internal capsule, basal ganglia, cerebellum; right cerebellum	Anomic Aphasia	Yes	Yes
**9**	F	66	14	1	75	Left unspecified	Not Aphasic by WAB	No	No
**10**	F	47	14	1	35	Left unspecified	Not Aphasic by WAB	No	Yes
**11**	F	74	16	1	40	Left frontal, insula, occipital, parietal, temporal; left basal ganglia, hippocampus	Conduction Aphasia	Yes	Yes
**12**	M	42	7	4	40	Left parietal, temporal; right cerebellum	Conduction Aphasia	Yes	Yes
**13**	M	79	18	1	61	Left occipital, parietal, temporal	Anomic Aphasia	Yes	Yes

**14**	M	77	14	1	60	Left frontal, insula, parietal, temporal; right parietal	Wernicke’s Aphasia	Yes	Yes
**15**	M	38	12	1	76	Left internal capsule, basal ganglia, thalamus, midbrain; Right frontal	Not Aphasic by WAB	Yes	Yes
**16**	M	49	14	1	23	Left frontal, insula, temporal; left amygdala, basal ganglia, hippocampus, thalamus	Anomic Aphasia	Yes	Yes
**17**	F	66	18	2	29	Left unspecified	Anomic Aphasia	Yes	Yes
**18**	M	58	16	1	114	Left frontal, insula, occipital, parietal, temporal; left amygdala, basal ganglia, hippocampus, thalamus	Conduction Aphasia	Yes	Yes
**19**	F	87	20	1	35	Left unspecified	Not Aphasic by WAB	No	Yes

Par – Participant; Edu – years of education; # of Strokes – number of strokes; TPO – time post stroke onset in months

Supplemental Table 2. Skew and Kurtosis values for each measure.

Control

		Eyes Open Rest				Eyes Closed Rest			
		**Delta**	**Theta**	**Alpha**	**Beta**	**Delta**	**Theta**	**Alpha**	**Beta**
**Whole Brain**	**Skew**	0.466	-0.893	3.121	0.352	0.855	0.368	0.444	0.302
	**Kurtosis**	-0.052	-0.117	11.861	-1.105	0.351	-0.231	-0.855	-0.509
**Clinical**	**Skew**	0.690	-0.874	2.949	0.222	0.845	0.565	0.468	0.117
	**Kurtosis**	0.540	0.152	10.665	-1.293	0.173	0.420	-0.737	-0.958
**Left Hemisphere**	**Skew**	0.585	-0.799	2.778	0.026	0.876	0.400	0.596	0.092
	**Kurtosis**	0.014	-0.125	9.711	-1.151	0.806	-0.195	-0.412	-1.070
**Right Hemisphere**	**Skew**	0.723	-0.862	3.299	0.621	0.915	0.232	0.393	0.439
	**Kurtosis**	-0.316	-0.213	12.928	-0.575	0.496	-0.648	-1.075	-0.238

Persons with Aphasia

		Eyes Open Rest				Eyes Closed Rest			
		**Delta**	**Theta**	**Alpha**	**Beta**	**Delta**	**Theta**	**Alpha**	**Beta**
**Whole Brain**	**Skew**	-0.482	0.297	1.704	-0.244	-0.528	0.638	1.554	-0.020
	**Kurtosis**	-0.361	-1.086	2.941	-0.324	-0.329	-0.821	1.868	-0.514
**Clinical**	**Skew**	-0.473	0.333	1.753	-0.295	-0.265	0.692	1.582	-0.028
	**Kurtosis**	-0.303	-1.038	3.117	-0.453	-0.124	-0.787	2.006	-0.534
**Left Hemisphere**	**Skew**	-0.540	0.390	1.338	-0.253	-0.677	0.489	0.755	0.263
	**Kurtosis**	0.237	-0.926	1.010	-0.805	0.258	-0.366	-0.057	-0.028
**Right Hemisphere**	**Skew**	-0.341	1.030	1.243	-0.285	-0.096	0.663	1.837	0.208
	**Kurtosis**	-1.391	0.717	1.162	-0.598	-0.973	-0.159	3.025	-0.386
